# The 21-gene Recurrence Score® assay predicts distant recurrence in lymph node-positive, hormone receptor-positive, breast cancer patients treated with adjuvant sequential epirubicin- and docetaxel-based or epirubicin-based chemotherapy (PACS-01 trial)

**DOI:** 10.1186/s12885-018-4331-8

**Published:** 2018-05-04

**Authors:** Frédérique Penault-Llorca, Thomas Filleron, Bernard Asselain, Frederick L. Baehner, Pierre Fumoleau, Magali Lacroix-Triki, Joseph M. Anderson, Carl Yoshizawa, Diana B. Cherbavaz, Steven Shak, Lise Roca, Christine Sagan, Jérôme Lemonnier, Anne-Laure Martin, Henri Roché

**Affiliations:** 1 Department of Biopathology, Centre Jean Perrin and EA 4677 ERTICa, Université d’Auvergne, 58 rue Montalembert, 63000 Clermont-Ferrand, France; 2 Department of Biostatistics, Institut Claudius Régaud, Institut Universitaire du Cancer Toulouse-Oncopole, Toulouse, France; 3 Department of Biostatistics, Institut Curie, Paris, France; 4 Genomic Health Inc, Redwood City, CA USA; 5 University of California, San Francisco, CA USA; 6 Department of Medical Oncology, Centre Georges François Leclerc, Dijon, France; 7 Department of Pathology, Institut Claudius Régaud, Institut Universitaire du Cancer Toulouse-Oncopole, Toulouse, France; 8 Department of Biostatistics, Centre Val d’Aurelle, Montpellier, France; 9 Department of Pathology, Institut de Cancérologie de l’Ouest (site René Gauducheau), Nantes, Saint-Herblain France; 10 Unicancer, Paris, France; 11 Department of Medical Oncology, Institut Claudius Régaud, Institut Universitaire du Cancer Toulouse-Oncopole, Toulouse, France

**Keywords:** Adjuvant chemotherapy, Breast cancer, Docetaxel, Epirubicin, Hormone receptor-positive, Lymph node-positive, Oncotype DX® 21-gene assay, Recurrence score® result, Tamoxifen

## Abstract

**Background:**

The 21-gene Recurrence Score (RS) result predicts outcome and chemotherapy benefit in node-negative and node-positive (N+), estrogen receptor-positive (ER+) patients treated with endocrine therapy. The purpose of this study was to evaluate the prognostic impact of RS results in N+, hormone receptor-positive (HR+) patients treated with adjuvant chemotherapy (6 cycles of FEC100 vs. 3 cycles of FEC100 followed by 3 cycles of docetaxel 100 mg/m^2^) plus endocrine therapy (ET) in the PACS-01 trial (*J Clin Oncol* 2006;24:5664-5671).

**Methods:**

The current study included 530 HR+/N+ patients from the PACS-01 parent trial for whom specimens were available. The primary objective was to evaluate the relationship between the RS result and distant recurrence (DR).

**Results:**

There were 209 (39.4%) patients with low RS (< 18), 159 (30%) with intermediate RS (18-30) and 162 (30.6%) with high RS (≥ 31). The continuous RS result was associated with DR (hazard ratio = 4.14; 95% confidence interval: 2.67-6.43; *p* <  0.001), adjusting for treatment. In multivariable analysis, the RS result remained a significant predictor of DR (p <  0.001) after adjustment for number of positive nodes, tumor size, tumor grade, Ki-67 (immunohistochemical status), and chemotherapy regimen. There was no statistically significant interaction between RS result and treatment in predicting DR (*p* = 0.79).

**Conclusions:**

After adjustment for clinical covariates, the 21-gene RS result is a significant prognostic factor in N+/HR+ patients receiving adjuvant chemoendocrine therapy.

**Trial registration:**

Not applicable.

## Background

Hormone-receptor–positive breast cancer accounts for 60 to 65% of all malignant neoplasms of the breast and the majority of these patients will receive endocrine therapy. Decisions on whether to offer adjuvant chemotherapy are assisted by identifying patients at greater risk of recurrence. Gene expression profiling augments information provided by traditional histopathologic factors and biomarkers by providing quantitative recurrence risk estimates. Today these technologies are frequently used for making treatment decisions in patients with early-stage breast cancer [1–6]. The Oncotype DX® 21-gene Recurrence Score (RS) assay predicts and quantifies the risk of DR, and overall survival (OS) in node-negative (N-)/node-positive (N+), estrogen receptor-positive (ER+) breast cancer patients treated with adjuvant endocrine therapy (ET) [5, 7]. The assay also predicts the magnitude of chemotherapy benefit in endocrine therapy treated N−/ER+ and postmenopausal, N+/ER+ patients [8, 9]. Additional studies (ECOG E2197, NSABP B-28, and PlanB) have demonstrated that RS results are predictive of the risk of recurrence and survival in patients who received adjuvant chemoendocrine therapy [10–12].

The PACS-01 trial was one of the landmark trials showing the benefit of adding a taxane to standard anthracycline adjuvant treatment in N+, early stage breast cancer [13, 14]. This study compared 6 cycles of FEC100 (6 cycles of 5-fluorouracil 500 mg/m^2^, epirubicin 100 mg/m^2^, cyclophosphamide 500 mg/m^2^), one of the standard treatments in the 1990’s [15, 16], to a sequential treatment of 3 cycles of FEC100 followed by 3 cycles of docetaxel 100 mg/m^2^ (FEC-D). After a median follow-up of 5 years, this study showed that the sequential arm significantly improved the disease-free survival (DFS) compared to FEC100 (78.4% vs. 73.2%, respectively) with a 17% relative reduction in the risk of relapse with FEC-D (p-adjusted for prognostic factors = 0.012). Five-year overall survival rates were 86.7% with FEC100 and 90.7% with FEC-D, demonstrating a 27% relative reduction in the risk of death (*p*-adjusted = 0.017). The multivariate analysis showed that FEC-D brought a significant benefit in postmenopausal women even in those with a low nodal involvement (i.e. 1 to 3 positive nodes). As molecular tools differentiate risk of recurrence in ET treated patients, a similar stratification of risk of recurrence in chemotherapy treated patients will help identify patients who remain at high risk of recurrence, e.g., “residual risk” in whom novel therapies such as CDK 4/6 inhibitors may be studied in order to improve outcomes in HR+ advanced breast cancer.

We evaluated the ability of the RS to predict the residual risk of DR and DFS for N+, hormone receptor-positive (HR+) women treated with adjuvant chemoendocrine therapy, from the PACS-01 trial.

## Methods

### PACS-01 trial design

The parent trial, PACS-01, was a randomized, multicenter, open-label phase III study conducted from June 1997 to March 2000 in 85 centers in France and Belgium, and enrolled 1999 women [13, 14]. Patients were assigned to receive FEC100 (5-fluorouracil 500 mg/m^2^, epirubicin 100 mg/m^2^, cyclophosphamide 500 mg/m^2^) intravenously on day 1 every 21 days for six cycles or the same regimen of FEC100 for three cycles followed by docetaxel 100 mg/m^2^ intravenously on day 1 every 21 days for three cycles (FEC-D). Stratification was by age (< 50 and ≥ 50 years), number of positive axillary lymph nodes (1 to 3, and > 3), and center.

Tamoxifen 20 mg/day was started after chemotherapy completion and continued for five years. Initially, tamoxifen was required for postmenopausal women with HR+ tumors. In December 1998, the study protocol was amended to also require tamoxifen treatment for premenopausal women with HR+ tumors. Post-lumpectomy radiotherapy was mandated. Post-mastectomy or regional-nodal radiotherapy was prohibited.

### Study population

**Fig. 1:**
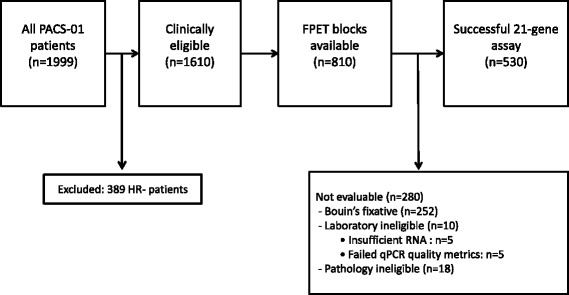
Modified REMARK diagram resulting in the final sample size of 530 patients. HR-, hormone-receptor negative; FPET, fixed paraffin embedded tumor; RNA, ribonucleic acid; qPCR, quantitative real-time polymerase chain reaction

Patients from the parent PACS-01 trial were eligible for enrollment in this study if they had N+/HR+ tumor as assessed by immunohistochemistry (IHC) with available formalin fixed paraffin embedded tumor (FPET), and successful 21-gene RS assessment (Fig. 1). Written informed consent was obtained from each patient in a standard procedure at each participating institution. The study was reviewed and approved by the Ethics Committee and Institutional Review Board of each participating site and was conducted according to the declaration of Helsinki and European Good Clinical Practice Requirements.

### 21-gene Recurrence Score assay

Available formalin fixed paraffin embedded tumor specimens from PACS-01 trial that met the above eligibility criteria had 5-μm tissue sections stained by hematoxylin and eosin (H&E) and centrally evaluated for histologic grade using the modified Bloom-Richardson score [14, 17]. All specimens were then analyzed by the 21-gene Oncotype DX® Recurrence Score Assay, as previously described [5, 16–19]. Critical preanalytical details include that every case has a representative H&E section reviewed by a Genomic Health, Inc. (GHI) board certified surgical pathologist with breast expertise and they mark the tumor-rich areas for manual-micro dissection, excluding biopsy cavities, skin and skeletal muscle. 30 μm of tissue were utilized unless tumor measured less than 1.0 cm where 60 μm were used. Bouin’s fixed samples, by chart review or visual inspection, were omitted from the analysis. RNA was extracted according to standard operating procedures for the 21-gene assay. The RNA was then assessed for quantity (Ribogreen assay™; Molecular Probes/Invitrogen; Eugene, OR), and confirmed free of residual genomic DNA (using a DNA-specific polymerase chain reaction [PCR] assay). RNA was subjected to reverse transcription (RT) with a universal RNA (Stratagene; Agilent; Santa Clara, CA) used as a positive control, and water as a negative control for each set of RT reactions followed by quantitative PCR analysis. The aggregate average reference gene expression served to normalize expression and as a quality metric for each sample, and the limit of detection and limit of quantitation cutoffs and other quality metrics, as defined for the 21-gene assay, was applied as pre-specified for the 21 genes in the RS result.

### Statistical considerations

In the parent trial, the primary endpoint was DFS, defined as the time from randomization to first relapse (local, regional, or distant) or death from any cause, and OS was a secondary endpoint defined as the time from randomization until death from any cause.

For this study, the RS assessment was pre-specified in the protocol and statistical analysis plan prior to assay processing. In this exploratory analysis, the primary objective was to assess the correlation between RS results and DR in N+/HR+ patients (time from random assignment to first DR; all other events were ignored). The secondary objectives were to determine an association between RS results and DR according to treatment (FEC100 or FEC-D), the correlation between RS results and clinicopathologic features, and to describe the distribution of RS value within subgroups of patients as defined by these features. The standard RS cut-offs were used: low (< 18), intermediate (18–30), and high (≥ 31) [5].

The Kaplan-Meier estimates and log-rank test were used to evaluate outcomes in the different RS groups. A Cox model was used to assess the strength of the association between continuous RS value (for an increase of 50 points) and outcomes, adjusting for treatment group. A multivariate Cox regression model, adjusted for classical clinicopathologic factors, was used to assess the independent prognostic impact of RS. A *p*-value < 0.05 was used to determine the statistical significance of findings.

## Results

### Patients’ characteristics

Of the 1999 patients participating in the parent PACS-01 trial, 389 were excluded from the current study because they had HR-negative tumors, leaving 1610 clinically eligible patients. Of those, tissue blocks were available for 810 patients. Of the 810 HR+ patients (ER+ and/or progesterone receptor-positive [PgR+]) with tumor blocks available, 530 were formalin fixed and eligible for the RS assessment (Fig. 1). Of those, 393 (74.2%) received tamoxifen.

The comparison between HR+ patients eligible (*n* = 530) and non-eligible (1080) for the RS assessment is presented in Tables 1, and 39.6% of eligible patients presented with at least 4 positive lymph nodes. Overall, the eligible patients had similar clinicopathologic features compared with the whole population of HR+ patients of the parent trial (Table 1). Compared with excluded patients, the eligible patients were significantly more likely to have high-risk features including less frequent PgR+ tumors (*p* <  0.001), and a higher rate of Ki-67 ≥ 14% (*p* = 0.003) [19]. Eligible patients were also more likely to have ER+ tumors (p = 0.003). Moreover, the eligible population showed a trend to be younger than 50 years and to have fewer ductal carcinomas. Median follow-up for DR was 7.7 years.

**Table 1:** Characteristics of hormone receptor-positive patients, eligible and non-eligible for RS assessment

Characteristics, *n* (%)	PACS-01 (*n* = 1610)	Eligible (*n* = 530)	Non-eligible (*n* = 1080)	*p*
FEC-D	820 (50.9)	268 (50.6)	552 (51.1)	0.84
Age ≥ 50 years	809 (50.2)	250 (47.2)	559 (51.8)	0.08
Mastectomy	708 (44.0)	215 (40.6)	493 (45.6)	0.053
≥ 4 positive nodes	595 (37.0)	210 (39.6)	385 (35.6)	0.12
Tumor size > 20 mm	701 (47.7)	244 (49.3)	457 (46.9)	0.28
Missing	141	35	106	
Ductal carcinoma	1204 (74.8)	381 (71.9)	823 (76.2)	0.06
SBR grade				0.31
Grade 1	216 (14.4)	76 (15.3)	140 (14.0)	
Grade 2	788 (52.5)	251 (50.4)	537 (53.6)	
Grade 3	496 (33.1)	171 (34.3)	325 (32.4)	
Missing	110	32	78	
Ki-67 expression ≥14% [19]	270 (36.7)	198 (40.5)	72 (29.1)	0.003
Missing	874	41	833	
HER2-positive	81 (9.7)	46 (8.8)	35 (11.3)	0.23
Missing	776	5	771	
Hormone receptor by IHC				
ER+	1493 (92.7)	506 (95.5)	987 (91.4)	0.003
PgR+	1186 (73.7)	355 (67.0)	831 (76.9)	< 0.001
ER+/PgR+	1069 (66.4)	331 (62.5)	738 (68.3)	< 0.001
ER+/PgR-	424 (26.3)	175 (33.0)	249 (23.1)	
ER-/PgR+	117 (7.3)	24 (4.5)	93 (8.6)	

*DFS* disease-free survival, *FEC-D* 3 cycles of FEC (6 cycles of 5-fluorouracil 500 mg/m^2^, epirubicin 100 mg/m^2^, cyclophosphamide 500 mg/m^2^) followed by 3 cycles of docetaxel 100 mg/m^2^, *FEC100* 6 cycles of 5-fluorouracil 500 mg/m^2^, epirubicin 100 mg/m^2^, cyclophosphamide 500 mg/m^2^, *SBR* Scarff–Bloom Richardson, *IHC* immunohistochemistry, *ER* estrogen receptor, *PgR* progesterone receptor

### Distribution of the Recurrence Score results

**Fig. 2:**
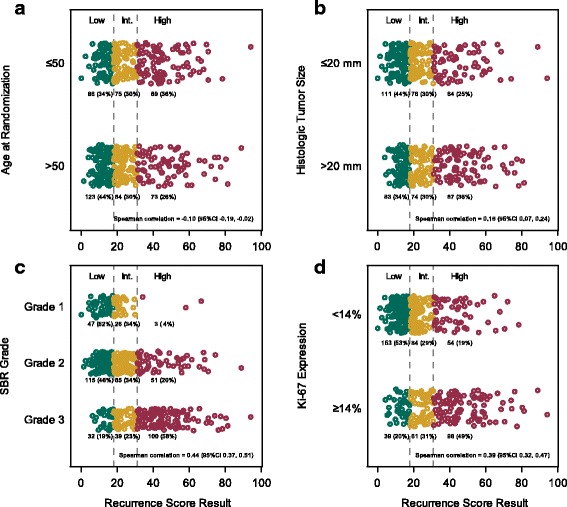
**a** Distribution of Recurrence Score results by (**a**) age, (**b**) tumor size, (**c**) tumor grade, and (**d**) Ki-67 expression

Among the 530 eligible patients, 209 (39.4%) had low RS results (< 18), 159 (30.0%) had intermediate RS results (18-30), and 162 (30.6%) had high RS results (≥ 31). The RS result was not significantly associated with the type of surgery (*r* [Spearman correlation] = 0.01; 95% confidence interval [95CI], − 0.07 to 0.10), or number of positive nodes (*r* = 0.06; 95CI: -0.02 to 0.15). There were statistically significant associations between RS results and age (*r* = − 0.10; 95CI, − 0.19 to − 0.02), tumor size (*r* = 0.16; 95CI, 0.07 to 0.24), tumor grade (*r* = 0.40; 95CI, 0.33 to 0.47), and Ki-67 expression (*r* = 0.39; 95CI, 0.32 to 0.47). Older patients, patients with small tumors, patients with lower tumor grade, and patients with Ki-67 expression < 40% were more likely to have a low RS result, although a wide distribution of RS values was seen in each age, tumor size, tumor grade, and Ki-67 category (Fig. 2). These results collectively demonstrate that the RS group cannot be simply predicted by nodal status or other clinicopathologic factors.

### Univariate analysis of outcomes according to Recurrence Score subgroups

In the protocol-specified primary analysis that adjusted for treatment, the continuous RS result was a statistically significant predictor of the risk of DR (hazard ratio [HR] = 4.14; 95CI: 2.67-6.43; *p* <  0.001), and DFS (HR = 3.28; 95CI: 2.18-4.94; p <  0.001).

**Fig. 3:**
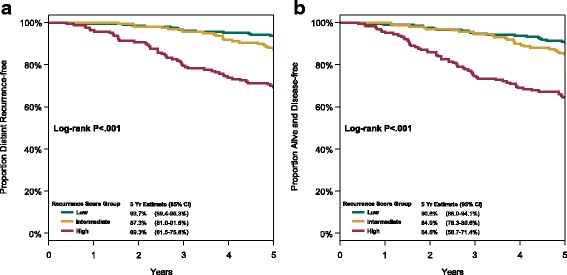
Disease outcome according to Recurrence Score group: (**a**) freedom from distant recurrence, and (**b**) disease-free survival

**Fig. 4:**
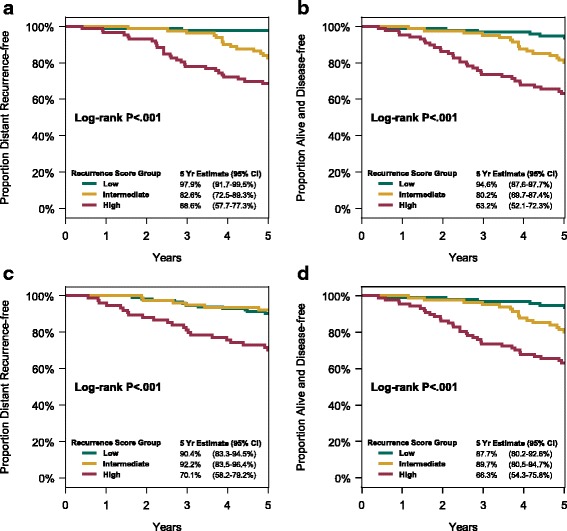
Disease outcome according to Recurrence Score group and chemotherapy treatment arm: (**a**) freedom from distant recurrence, (**b**) disease-free survival in patients treated with FEC100, (**c**) freedom from distant recurrence, and (**d**) disease-free survival in patients treated with FEC-D

Disease outcome was significantly associated with RS groups for DR, DFS, and OS (Table 2, Fig. 3). This association was not influenced by chemotherapy treatment arm (Fig. 4), and there was no statistically significant interaction between RS results and treatment in predicting DR (*p* = 0.79) or DFS (*p* = 0.61).

**Table 2:** Association between Recurrence Score results and disease outcomes

	5-year Kaplan-Meier risk estimate (%) and 95% CI by Recurrence Score group			
Risk	Low (< 18)	Intermediate (18-31)	High (≥ 31)	HR per 50 units^*^ (95% CI)
DR	93.7 (89.4-96.3)	87.3 (81.0-91.6)	69.3 (61.5-75.8)	4.14 (2.67-6.43)
	*p* < 0.001^†^			*p* < 0.001^§^
DFS	90.8 (86.0-94.1)	84.9 (78.3-89.6)	64.6 (56.7-71.4)	3.28 (2.18-4.94)
	*p* < 0.001^†^			*p* < 0.001^§^
OS	99.0 (96.2-99.8)	95.6 (90.9-97.9)	85.6 (79.1-90.2)	5.0 (3.01-8.28)
	*p* < 0.001^†^			*p* < 0.001^§^

*DR* distant recurrence, *95%CI* 95% confidence interval, *HR* hazard ratio, *DFS* disease-free survival, *OS* overall survival

^*^Recurrence Score was a continuous variable in a Cox proportional hazards regression model, with the HR adjusted for treatment and calculated relative to an increment of 50 units

^†^*p*-value from log-rank test for comparison of entire Kaplan-Meier plots among the three Recurrence Score groups

^§^p-value from Wald test for significance of HR

### Multivariable analysis of outcomes adjusted for clinicopathologic factors

The multivariable analysis adjusted for prognostic factors showed that continuous RS result, number of positive lymph nodes and pathological tumor size were independent prognostic factors of DR and DFS (Table 3). Additionally, endocrine therapy was an independent prognostic factor of DFS.

**Table 3:** Cox regression model analysis for distant recurrence and disease-free survival

Variable	HR (95%CI)	*p*
DR		
Recurrence Score (50 unit)	3.36 (1.88-6.00)	< 0.001
FEC-D	1.15 (0.75-1.75)	0.529
≥ 4 positive nodes	2.68 (1.73-4.17)	< 0.001
Tumor size > 20 mm	1.76 (1.12-2.78)	0.015
SBR grade		0.280
Grade 2	2.29 (0.81-6.48)	
Grade 3	2.32 (0.79-6.77)	
Ki-67 expression	1.19 (0.75-1.88)	0.453
DFS		
Recurrence Score (50 unit)	2.66 (1.62-4.37)	< 0.001
FEC-D	1.10 (0.76-1.59)	0.598
≥ 4 positive nodes	2.65 (1.82-3.87)	< 0.001
Tumor size > 20 mm	1.55 (1.06-2.26)	0.024
Endocrine therapy	0.60 (0.40-0.89)	0.012
Ki-67 expression	1.17 (0.79-1.74)	0.431

*HR* hazard ratio, *95CI* 95% confidence interval, *DR* distant recurrence, *FEC-D* 3 cycles of FEC100 (6 cycles of 5-fluorouracil 500 mg/m^2^, epirubicin 100 mg/m^2^, cyclophosphamide 500 mg/m^2^) followed by 3 cycles of docetaxel 100 mg/m^2^, *SBR* Scarff–Bloom Richardson, *DFS* disease-free survival

### Prognostic impact of Recurrence Score results

**Fig. 5:**
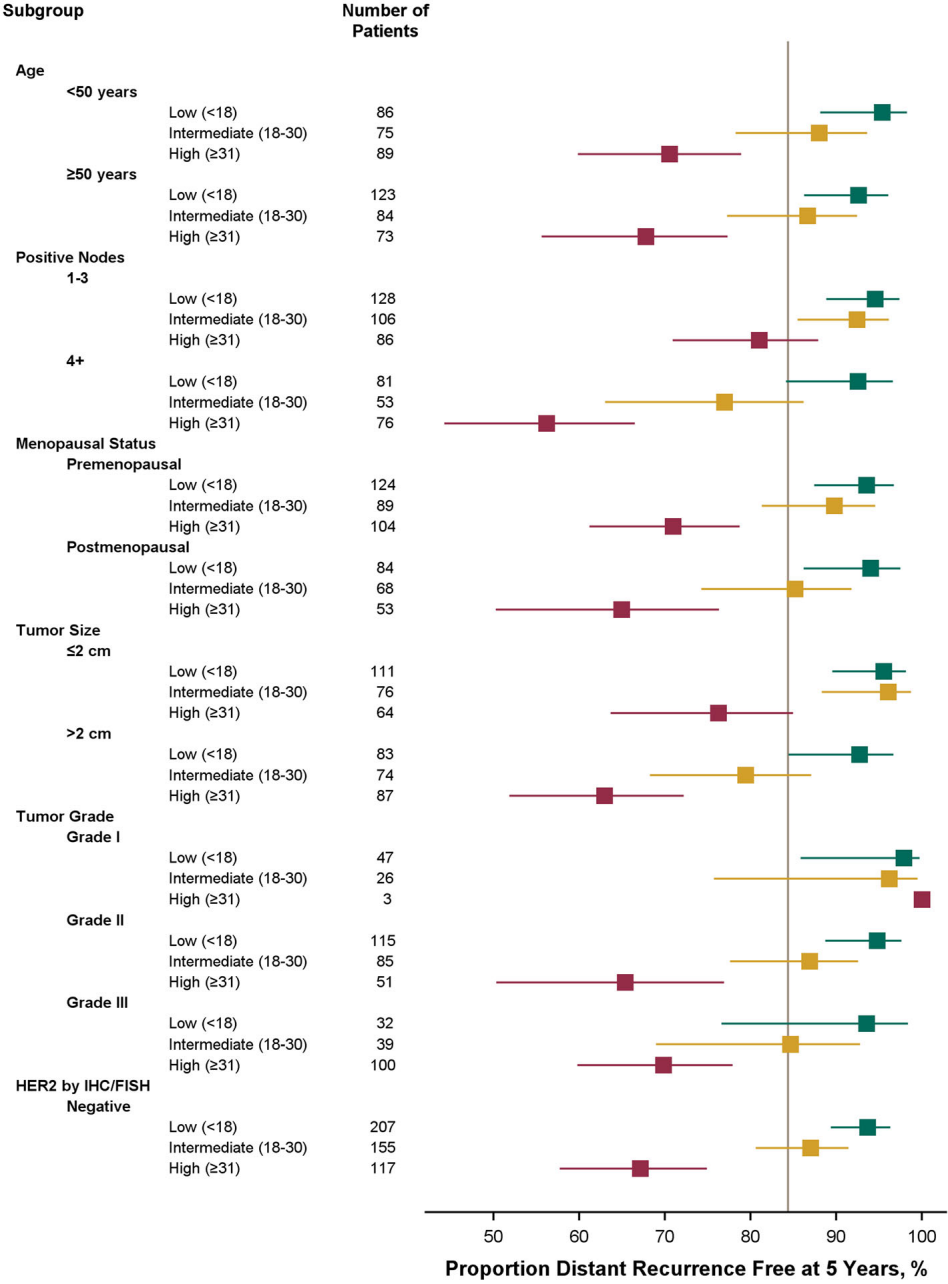
Forest plot of 5-year distant recurrence according to prognostic factors

The consistency of the prognostic impact of the RS result was evaluated by calculating Kaplan-Meier estimates of 5-year risks for DR by RS group in various patient subgroups defined by classical prognostic factors (Fig. 5). The prognostic impact was consistent across age at study entry, number of positive nodes, menopausal status, tumor size, and SBR grade; and maintained in patients with ER-positive and HER2-negative tumors. At 5 years, among 128 patients with 1-3 positive nodes and low RS results, only 9 (5.5%) had a DR event. Generally, outcomes of patients with ≥4 positive nodes were less favorable than those in patients with 1-3 positive nodes, but outcomes in patients with ≥4 positive nodes and low RS results were as good as or better than those of patients with 1-3 positive nodes and intermediate or high RS results (Fig. 5).

## Discussion

This study has shown that the 21-gene RS result was an independent predictor of outcome in N+/HR+ breast cancer patients treated with adjuvant chemoendocrine therapy. Multiple studies (ECOG E2197, NSABP B-28, and WSG PlanB) have now shown that the RS result can predict patients at residual risk of recurrence in N+ breast cancer patients treated with adjuvant chemoendocrine therapy [10–12, 18]. In the ECOG E2197 trial, the RS result was found to be independently prognostic in a cohort of 465 ER+, N- or N+ (1-3 involved lymph nodes) patients treated with doxorubicin plus cyclophosphamide (AC), or doxorubicin plus docetaxel (AT) [10]. In the NSABP B-28 trial, the RS result was independently prognostic in 1065 N+ patients treated with AC or AC followed by paclitaxel [11]. Our study confirms the B-28 prognostic results, that the RS predicts the residual risk of recurrence in patients with both 1-3 and > 3 positive lymph nodes, treated with chemoendocrine therapy. The RS was not predictive of taxane benefit.

Our findings suggest that gene expression as assessed by the RS provided independent prognostic information within clinically relevant nodal subgroups (N1 and N2). Patients with at least 4 positive lymph nodes and low RS results had outcomes as good as or better than patients with 1-3 positive lymph nodes and intermediate- or high-RS results. These findings are consistent with those reported from previous studies in N+ patients treated with ET alone [6, 8], and underscore the independent prognostic significance of tumor biology assessed by the RS in women with ER+ N+ breast cancer treated with chemoendocrine therapy. Furthermore, the RS is the only assay that has been shown to be predictive of late distant recurrence in ER+, N+ patients treated with chemoendocrine therapy [19]. The significance of these finding is important for clinical trial design as it allows for the inclusion of high risk N1 patients.

In the parent study, the treatment benefit with FEC-D was superior to FEC100 in postmenopausal, but not in premenopausal women [13]. Earlier studies, however, had shown that adjuvant chemotherapy provides a greater benefit in terms of DFS and OS for premenopausal than for postmenopausal women [20, 21]. In our study, the performance of the RS result was similar in pre- and postmenopausal women. This finding is consistent with the data presented from the NSABP B-28 study, providing support for RS assay use in premenopausal, N+ women [11]. Our study also showed a consistently strong association between the RS result and outcomes in each treatment arm.

The addition of taxanes to anthracycline-based chemotherapy regimens has resulted in a significant reduction in the odds of recurrence and death (approximately 15%-20% relative reduction in risk) [21–24]. This relative risk reduction has translated to an absolute improvement in DFS and OS of about 4%-5%. A similar benefit was observed in the NSABP B-28 trial, although no significant interaction between hormone receptor status and treatment was observed in this study that compared treatment with AC and AC followed by paclitaxel [11]. As in that study, we did not observe significant interaction between treatment and RS result for adding docetaxel to FEC100.

At the outset of the PACS-01 parent study, adjuvant tamoxifen was mandated for postmenopausal but not premenopausal women with HR+ tumors. Subsequently, a mid-study protocol amendment was implemented that mandated adjuvant tamoxifen for all women with HR+ tumors. As a result, approximately one quarter of the HR+ patients in this study were not treated with adjuvant hormonal therapy. One potential limitation of this study, therefore, could be inconsistent adjuvant hormonal therapy use in the analysis cohort. A second limitation was that HER2+ patients did not receive adjuvant anti-HER2 treatment as this trial was conducted in the pre-trastuzumab era. However, sensitivity analyses were performed on an exploratory basis to evaluate the consistency of the performance of the Recurrence Score in various subsets and in the 470 patients who were HER2-negative by IHC/FISH and the prognostic ability of the Recurrence Score was maintained. Another potential limitation of the current study is the exclusion of samples that were fixed in Bouin’s solution (*n* = 252). The decision to exclude these samples was made prior to unblinding of the clinical data, which minimized potential bias in sample selection.

Strengths of this study include the randomized controlled design of the parent trial, the high quality of central pathology, and the consistency of results for secondary clinical endpoints and across subgroups. Importantly, although all patients in this study received chemotherapy, the finding of few DR events in patients with 1-3 nodes and RS <  18 affirms the value of the 21-gene RS assay in N+, HR+ patients. The recently published results of the West German Study Group’s PlanB study showed that patients with pN0 or pN1 disease and RS < 12 have 3-year DFS of 98.4% (95%CI: 97.0%-99.8%) [12]. Our current study findings add to a growing body of supporting evidence that RS results provide an independent assessment of risk that can guide the selection of optimal therapy for patients with 1-3 positive lymph nodes. Finally, a recent analysis, performed by the Surveillance, Epidemiology, and End Results (SEER) Program, showed that, in more than 44,000 patients with N- and N+ disease (4691 patients with positive lymph nodes [N+(mic,1-3)]), the 21-gene assay accurately predicts prospective outcomes, independent of patient age, tumor size, and tumor grade [25]. Understanding in which patient populations a molecular assay has been validated is critical for their appropriate use in patient treatment decision making. Ideally a molecular assay will have shown consistency in predicting clinical outcome across diverse breast cancer populations. Determinations of extended endocrine therapy or whether to include a high-risk patient in a clinical trial often involve patients who have received chemotherapy. Molecular assays may have been shown to be strongly prognostic in breast cancer patients receiving endocrine therapy alone but not in those who have received chemotherapy [26].

## Conclusions

In conclusion, our results demonstrate that the Recurrence Score result is prognostic in node positive, HR+ patients treated with chemoendocrine therapy in a randomized clinical trial. It was not predictive of docetaxel benefit. The Recurrence Score result provides significant prognostic information beyond what traditional clinicopathologic features can provide in N1 and N2 patients, with similar performance with or without the addition of a taxane to standard chemotherapy. Noteworthy, the PACS-01 trial results were fully confirmed after an 8-year follow-up with a significant superiority of FEC-D over FEC100 [27]. Our findings showed that RS results help to identify subgroups of patients with low residual risk of recurrence that could be adequately treated with standard adjuvant treatment regimens. Importantly, RS results could also help to identify patients with considerable residual risk of recurrence who could be candidates for more aggressive adjuvant chemotherapy regimens and for participation in adjuvant trials evaluating novel agents such as CDK4/6 inhibitors.

## Abbreviations

95CI: 95% confidence interval
AC: Doxorubicin-cyclophosphamide
AT: Doxorubicin-docetaxel
DFS: Disease-free survival
DNA: Deoxyribonucleic acid
DR: Distant recurrence
ECOG: Eastern Cooperative Oncology Group
ER+: Estrogen receptor-positive.
ET: Endocrine therapy
FEC100: 6 cycles of 5-fluorouracil 500 mg/m^2^, epirubicin 100 mg/m^2^, cyclophosphamide 500 mg/m^2^
FEC-D: 3 cycles of FEC100 followed by 3 cycles of docetaxel 100 mg/m^2^
FPET: Fixed paraffin embedded tumor
GHI: Genomic Health, Inc.
H&E: Hematoxylin and eosin
HR: Hazard ratio
HR+: Hormone receptor-positive.
IHC: Immunohistochemistry
N-: Node-negative
N+: Node-positive.
NSABP: National Surgical Adjuvant Breast and Bowel Project
OS: Overall survival
PCR: Polymerase chain reaction
PgR+: Progesterone receptor-positive.
RNA: Ribonucleic acid
RS: Recurrence Score
RT: Reverse transcription
SBR: Scarff Bloom Richardson
